# Singleton mutations in large-scale cancer genome studies: uncovering the tail of cancer genome

**DOI:** 10.1093/narcan/zcae010

**Published:** 2024-03-12

**Authors:** Sanket Desai, Suhail Ahmad, Bhargavi Bawaskar, Sonal Rashmi, Rohit Mishra, Deepika Lakhwani, Amit Dutt

**Affiliations:** Integrated Cancer Genomics Laboratory, Advanced Centre for Treatment, Research, and Education in Cancer, Kharghar, Navi Mumbai 410210, Maharashtra, India; Homi Bhabha National Institute, Training School Complex, Anushakti Nagar, Mumbai 400094, Maharashtra, India; Integrated Cancer Genomics Laboratory, Advanced Centre for Treatment, Research, and Education in Cancer, Kharghar, Navi Mumbai 410210, Maharashtra, India; Homi Bhabha National Institute, Training School Complex, Anushakti Nagar, Mumbai 400094, Maharashtra, India; Integrated Cancer Genomics Laboratory, Advanced Centre for Treatment, Research, and Education in Cancer, Kharghar, Navi Mumbai 410210, Maharashtra, India; Integrated Cancer Genomics Laboratory, Advanced Centre for Treatment, Research, and Education in Cancer, Kharghar, Navi Mumbai 410210, Maharashtra, India; Integrated Cancer Genomics Laboratory, Advanced Centre for Treatment, Research, and Education in Cancer, Kharghar, Navi Mumbai 410210, Maharashtra, India; Integrated Cancer Genomics Laboratory, Advanced Centre for Treatment, Research, and Education in Cancer, Kharghar, Navi Mumbai 410210, Maharashtra, India; Integrated Cancer Genomics Laboratory, Advanced Centre for Treatment, Research, and Education in Cancer, Kharghar, Navi Mumbai 410210, Maharashtra, India; Homi Bhabha National Institute, Training School Complex, Anushakti Nagar, Mumbai 400094, Maharashtra, India; Department of Genetics, University of Delhi, South Campus, New Delhi 110021, India

## Abstract

Singleton or low-frequency driver mutations are challenging to identify. We present a domain driver mutation estimator (DOME) to identify rare candidate driver mutations. DOME analyzes positions analogous to known statistical hotspots and resistant mutations in combination with their functional and biochemical residue context as determined by protein structures and somatic mutation propensity within conserved PFAM domains, integrating the CADD scoring scheme. Benchmarked against seven other tools, DOME exhibited superior or comparable accuracy compared to all evaluated tools in the prediction of functional cancer drivers, with the exception of one tool. DOME identified a unique set of 32 917 high-confidence predicted driver mutations from the analysis of whole proteome missense variants within domain boundaries across 1331 genes, including 1192 noncancer gene census genes, emphasizing its unique place in cancer genome analysis. Additionally, analysis of 8799 TCGA (The Cancer Genome Atlas) and in-house tumor samples revealed 847 potential driver mutations, with mutations in tyrosine kinase members forming the dominant burden, underscoring its higher significance in cancer. Overall, DOME complements current approaches for identifying novel, low-frequency drivers and resistant mutations in personalized therapy.

## Graphical Abstract

**Graphical Abstract:**
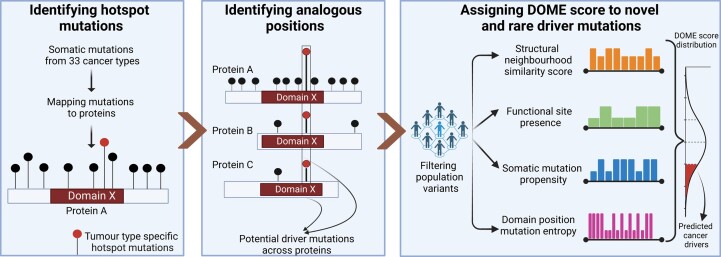

## Introduction

Singleton mutations are mutations that occur in a single individual or a small number of individuals within a cohort of cancer samples. Due to their low frequency, they are considered rare and insignificant. Recent studies, however, have highlighted their function in tumor development and progression (1,2). Due to their heterogeneity and complexity, identifying and predicting their functional impact in large-scale cancer genome studies presents significant challenges (3,4). Their rarity necessitates large sample sizes for confident detection, which increases computational and statistical burdens (5). Similarly, the identification of variants of unknown significance (VUS) poses a significant challenge to personalized cancer diagnostics as their effects on protein function and therapeutic targeting are unknown (6). Creating accurate computational methods to identify and prioritize singleton mutations is crucial for advancing cancer biology understanding and finding potential targets for personalized cancer therapy.

Proteome-based studies have reported specific domains to be enriched across tumor types, wherein an understanding of domain architecture provides better insights into the function of proteins (7). Also, conserved residue information within domains has been exploited to derive significance of somatic mutations and nominate domain hotspots (8,9). Resources exploiting the domain-centric data models of the somatic mutations have been useful in exploratory analysis (10,11). A pan-cancer mutation analysis of over 5000 patient samples across 33 tumor types has been the most systematic effort to catalog the domain restricted hotspots (12). The study revealed many interesting, rare gene mutations that were anonymous to alternative methods.

We present a framework for analyzing somatic mutations and predicting rare tumor driver events. We identify statistical hotspots by mapping TCGA (The Cancer Genome Atlas) somatic mutations for 33 cancer types onto the human proteome. Combining these locations with previously reported recurrent mutations and therapy-resistant mutations, we identify analogous domain family positions. We evaluate the functional significance of these variants using CADD scores and comparing them to known cancer-causing and benign variants. We propose a scoring scheme integrating somatic mutation propensity, domain-wide mutation distribution and residue context using protein structures. We identify known and novel nonrecurrent putative driver mutations by reanalyzing TCGA and 224 in-house tumors for seven cancer types. Our scoring framework is compared to existing methods, and a user-friendly toolkit, domain driver mutation estimator (DOME), is provided for simple access and downstream prioritization of somatic mutations.

## Materials and methods

### Protein sequences, domain, functional sites and domain interaction interface data

The reviewed human proteome sequences (*n* = 20,365) and protein annotations were downloaded from the UniProt database (version 2020_01) (13). This file was cross-referenced with the protein family database (PFAM, version 32.0) (14), consisting of 17,929 families. The fasta and swissprot format file parsing of protein features and sites was performed using Biopython-based in-house scripts (15). The post-translational modification sites were obtained from the PhosphoSitePlus database (16). Protein domain interface positions were obtained from the 3did database (17). The mapped protein positions mentioned in the above-mentioned databases (UniProt, PhosphoSitePlus and 3did) are termed as *functional sites*. To compute 3D geometric closeness between the amino acid positions across the proteins in the proteome, structures from the AlphaFold database (18) were obtained in PDB format and parsed through the in-house scripts to generate a residue network for individual proteins. Pairs of residues are considered as geometrically close (connected) if the distance between the two is <10 Å [based on (19)]. The distance-based network for the 3D structures was built using the NetworkX Python package.

### TCGA tumor mutation assignment to protein

The tumor-wise somatic mutation MAF files were obtained from TCGA FireBrowse (http://firebrowse.org/). Only the mutations from primary tumor samples were retained and metastatic/recurrent sample mutations (identified based on the TCGA barcode ID) were excluded from the analysis. Only the missense mutation entries with reviewed UniProt protein ID were included in the study. In total, the mutation data were obtained from 8575 tumor samples across 33 tumor types. Further, the mutations not mapping to the canonical protein from the UniProt or having residue position beyond the length of canonical protein were excluded. Residue-wise mutation counts for individual proteins were generated using in-house Python scripts.

### Statistical hotspot and therapy resistance mutations across different cancer types

Mutation hotspot analysis was performed for proteins across the human proteome, for each tumor type separately as well as using a pan-cancer cohort [MC3 (multi-center mutation calling in multiple cancers) mutations from TCGA]. With lower background variants in the gene, even low-frequency mutations would be nominated as hotspots, introducing false-positive drivers in the initial list. Hence, genes with <1% mutation recurrence within a particular tumor type were ignored from the analysis. With the tumor-wise mutation analysis using a binomial test, we identified 3298 significantly mutated positions [false discovery rate (FDR) < 0.05]. A protein/domain-based binomial distribution model was used, in which a residue was defined as a *hotspot* if it harbored more mutations than expected by chance:


(1) \begin{eqnarray*}{{{P}}}_{({{m}} = {{k}})} = {{C}}\left( {{{n}},{{k}}} \right){{{p}}}^{{k}}{\left( {1 - {{p}}} \right)}^{{{n}} - {{k}}},\end{eqnarray*}

where *C*(·) denotes the binomial coefficient, *n* is the total number of mutations in a protein and *k* is the mutation count at a residue whose significance is being tested. Since the assumption is that every residue has an equal likelihood of mutation, *p* is defined as 1 divided by total length of the domain or protein. The *P*-values were further corrected using the Bonferroni method. Tests were also performed separately on mutation count data from individual cancer types and statistical significance was computed for each mutation per tumor type. To reduce the false-positive hotpot positions, only the mutations occurring within the genes under selection in cancer [reported in (20)] were selected for further analysis. The following list was combined with hotspot mutation catalog reported at cancerhotspots.org, identified in 24,592 tumor samples by earlier reported methods (21,22) and the recurrent mutations reported in (21). Additionally, the therapy resistance-associated mutations reported in the COSMIC database (23) were also included, forming the core significant cancer mutations (*n* = 1527) (Supplementary Table S1).

### Derivation of analogous mutations across domains

Protein domain boundaries were obtained from the PFAM database and multiple sequence alignment for individual domains (within the human proteome) was generated using MAFFT aligner (24). The significant cancer mutations were mapped across the proteome and mutations falling outside the domain were ignored for the analogous mutation derivation. For the significant mutations falling within the domain, all the residues conserved (based on biochemical property—positively charged, negatively charged, polar, nonpolar and aromatic) in the aligned position (column in alignment) are termed ‘analogous’ to each other.

### Scoring of the somatic mutations within domain

**Figure 1.**
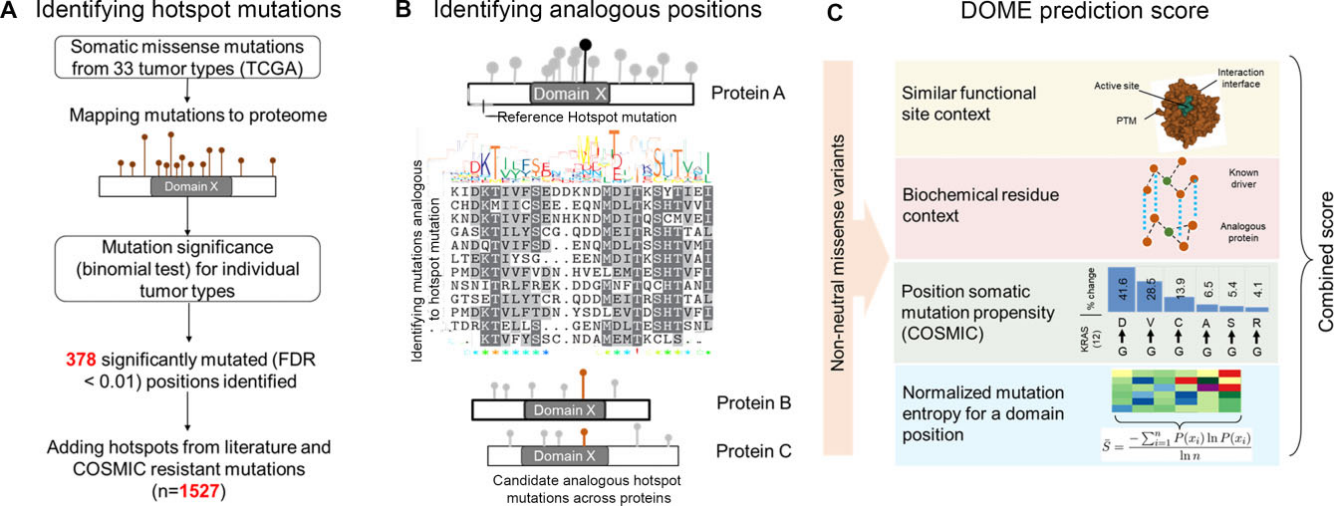
Workflow of the DOME algorithm to nominate the low-frequency driver mutations. (**A**) Missense somatic mutations found across 33 cancer types in TCGA are mapped to protein residues and evaluated based on recurrence for the statistical significance using a binomial test. Combining with the literature reported mutations [Cancer Hotspots database, recurrent mutations reported in (21) and resistant mutations in the COSMIC database] formed the hotspot/resistant mutation database consisting of 1527 mutation positions. (**B**) Protein A is shown to contain a hotspot mutation (black bar) within a functional domain (domain X). Below the protein, a multiple sequence alignment of the domain X sequences across proteins is shown (which includes sequence alignment across protein A and other members of the domain family; e.g. proteins B and C are shown below). For the significant positions (highlighted bar) occurring within the domain regions, analogous (aligned positions in domain) positions in proteins (e.g. proteins B and C) having the conserved domain residues are retrieved (colored highlighted bars). (**C**) The non-neutral (variants having potential phenotypic effect) positions (functional CADD score >20) within the analogous ones are scored based on the residue, biochemical context, mutation entropy across column within the domain and amino acid alteration frequency within the COSMIC database.

Individual somatic mutations (if found analogous) are checked for their non-neutral status based on an aggregated CADD score (25). CADD score for an individual position is defined as a mean of the CADD scores for all the possible amino acid variants (biochemically distinct) at a position. This mean CADD score is henceforth termed as *functional score* for a particular position. Lower CADD score (<10) indicates the germline status of the variant, whereas CADD score >20 is considered functionally deleterious (non-germline in nature). Hence, somatic mutations having functional CADD score >20 were further selected for scoring. Each of the analogous mutation obtained (as described previously) is scored based on (i) functional context—defined by the presence of PTM/active/protein interface site, similar to the statistical significant mutation, (ii) biochemical residue context—similarity of the proximal biochemical environment of the mutant protein position to the known hotspot/resistant mutation, (iii) somatic mutation propensity for that mutation, as reported in COSMIC, and (iv) normalized mutation entropy (NEM) for the domain position [as computed in (12)]. In brief, NEM is defined by the following equation:


(2) \begin{eqnarray*}\mathop S= {- \sum \limits_{i = 1}^n P\left( {{x}_i} \right)\ln P\left( {{x}_i} \right)\over \ln n} \end{eqnarray*}

where *S* is the normalized entropy, *n* is the number of residues aligned within a column of multiple sequence alignment (within a domain) and *x_i_* is the number of mutations observed at *i*th element within the array (*n*) of residues across proteins within the domain. The value of NEM or *S* is within 0–1 range. A cumulative score, ranging from 0 to 1, is ascribed to individual somatic mutation, based on the mean of the scores (i–iv). This cumulative score is used to rank the somatic mutations found analogous to the known hotspot/resistant mutations. The overview of the analysis framework is shown in Figure 1.

### Benchmarking of DOME with other driver detection methods

The missense somatic mutations from TCGA (MC3) were used for benchmarking of DOME results. Primarily annotated missense variants, CTAT (combined tool adjusted total) predictions and predicted outcomes for variants from other algorithms used for benchmarking in the study by Bailey *et al.* (1) were downloaded from the GDC (https://gdc.cancer.gov/about-data/publications/pancan-driver). Only those mutations that were within the domain and overlapping with the analogous positions of the hotspot mutations formed the denominator for benchmarking. Two linear tracks (functional and cancer driver) provided with the missense mutations were used as truthset. CTAT scores (specifically CTAT cancer scores) were tested against the linear truthset annotations (tracks) and area under the receiver operating characteristic curve (AUC) was found to be 0.94. Further, we used six methods reported in the study by Bailey *et al.* (1): SIFT (26), MutationTaster (27), FATHMM (28), MutationAssessor (29), and PolyPhen-2 HDIV and HVAR (30). These methods were selected since their scoring systems are based on the biological information of variants and not on training datasets based on cancer-associated somatic mutations (similar to DOME). Scores from all the methods were directly compared against the truthset tracks and AUC was analyzed using the pRUC (31).

To evaluate the overlap of predictions of DOME with other tools, whole set of missense mutations from the MC3 mutation set (TCGA harmonized variant calls) found within the protein domains and at analogous positions was used.

These mutations were scored using the DOME framework and also queried through the dbNSFP database (32). From the database, seven different tools were utilized for benchmarking: six are mentioned earlier (SIFT, MutationTaster, FATHMM, MutationAssessor, PolyPhen-2 HDIV and HVAR) and additionally PROVEAN (33) was used, since it works on protein sequence homology (conservation)—a method comparable in the segment to DOME (26,27,33–36).

Mutation positions with positive scores were classified as deleterious (D), while the rest were considered tolerant (T). Predictions from seven tools were categorized as ‘D’ or ‘T’ based on their defaults. For tools with multiclass predictions, ‘D’ or ‘T’ was assigned depending on functional impact. Jaccard coefficient was used to compare individual mutation classifications. Similarly, to nominate the driver gene ranks given for the comparison of top gene lists (10, 20, 50 and 100) were based on the number of unique mutations predicted to be deleterious within each gene. Additionally, resources like DriverDB3 (37), ClinVar (38) and OncoKB (39) and literature search were used to identify known driver mutations from DOME analysis.

### Development of the resource databases and interface to access DOME

The DOME algorithm is implemented in Python using the data processing libraries Pandas and Numpy. The graphical user interface (GUI) is created using RShiny. It generates a database of analogous positions across domains by comparing 20,380 human proteome proteins to cancer-associated mutations, functional sites and related positions using NetworkX. The results are stored in tabix-indexed text-file-based databases (40). A GUI-based toolkit facilitates the analysis of somatic mutation datasets and facilitates access to proteome-based analysis results. The details about usage of the toolkit have been summarized in the user manual (available at https://www.actrec.gov.in/pi-webpages/AmitDutt/DOME/DOME.html).

### Somatic variant analysis of in-house tumor exome samples

In total, 224 in-house whole-exome tumor samples (180 paired and 44 tumor only), representing seven tumor types were analyzed using the GATK (v. 4.1.8) (41) variant analysis pipeline, as previously described (42). The seven tumor type samples analyzed for somatic variant detection were breast (*n* = 43), gall bladder (*n* = 17), cervical (*n* = 33), lung (*n* = 52), tongue (*n* = 24), colorectal (*n* = 27) and thyroid (*n* = 48). A standard GATK-based variant calling pipeline was used. In short, alignment to GENCODE reference genome (GRCh38.p12) (43) was performed using BWA-mem aligner (44), followed by GTAK preprocessing and HaplotypeCaller-based variant calling, followed by hard filtering, as previously described (42). Additionally, somatic calls were generated using Mutect2 (v4.2.3) from the tumor samples with matched normal (*n* = 173) and tumor-only modes using a panel of normals (PON). Variants failing the quality filters (base_qual, map_qual, position, strand_bias) and out of the defined target capture region (defined by individual capture kit) were removed. Subtraction of paired normal variants was performed for GATK variant calls as well; finally, somatic variant calls obtained from GATK and Mutect2 were combined. The somatic variants were subject to functional annotation using the VEP tool (45). The variants were annotated using in-house scripts with the germline databases, including gnomAD (46), dbSNP (47), TMC-SNPdb 2.0 (42), in-house PON and COSMIC (23).

## Results

### Overview of the DOME analysis framework and functional relevance of the analogous positions

The DOME framework extends upon the biological information that domains encode conserved structural modules, involved in specific molecular functions, which may be duplicated across the functionally related genes. Mutations in such segments at similar positions (or hereafter termed *analogous positions*) may exert similar functional impact, especially in the case of oncogenic driver mutations. To identify putative driver mutations from such analogous positions, missense somatic mutations (*n* = 827,343) from 7541 patient samples representing 33 tumor types from TCGA were mapped to the human proteome. Of these, 45.5% (*n* = 376,513) mutations mapped to conserved PFAM domains, consistent with earlier reports (48). Genes having a mutation frequency of >1% in any tumor type were used to identify hotspot mutations across different cancers. The median number of unique genes bearing at least a mutation was 1541 per tumor. Statistical significance for individual protein position across the human proteome was computed for each cancer type using a binomial model, as described earlier (12). To reduce the false positives, mutations within the genes reported to be under selection in somatic tissue, as per study (20), were used further. Analysis identified 378 statistically significant (FDR < 0.05) hotspot mutations across all the tumor types. Additionally, cancer hotspot mutations reported in the recurrent analysis (21) of TCGA data and cancerhotspots.org database were included in the study to create a DOME reference hotpot mutation dataset, consisting of 1240 unique mutations across 401 proteins. Majority of the hotspot genes and mutations were found to be overlapping across the three resources, with the binomial analysis contributing to 26 additional mutations (Supplementary Figure S1). Additionally, cancer-resistant mutations are also known to show evolutionary constraint and conservation across domains (49); hence, resistant mutations from the COSMIC database were added to the hotspot mutation list, resulting in 1527 unique mutations (Supplementary Table S1). Of the 1527 mutation positions, 991 were found to be within *pfam* domain boundaries. These 991 positions were probed within the domain alignments to identify the positionally aligned, biochemically conserved residues (as described in the ‘Materials and methods’ section), wherein 32,099 positions were obtained spanning across 83 domains.

**Figure 2.**
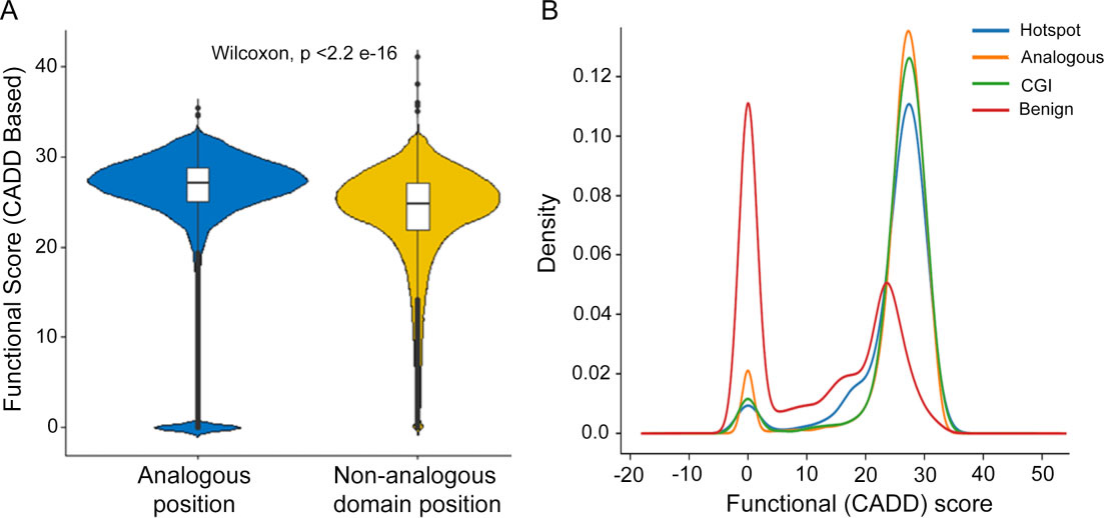
CADD functional score distribution of the analogous positions within the human proteome. (**A**) Comparison of CADD scores across analogous and non-analogous positions within the human protein domains. (**B**) CADD score distribution of the cancer hotspots identified by DOME (hotspot), curated driver mutations from the Cancer Genome Interpreter (CGI) and benign variants from ClinVar (benign), compared to the analogous protein sites (analogous) identified by DOME.

We evaluated whether the analogous positions showed an enrichment of *functional score* (described in the ‘Materials and methods’ section) with respect to the background scores of the domain positions (non-analogous) within same proteins. The analogous positions were observed to have significantly higher functional score with respect to the background positions (*P*-value <2.2 × 10^−16^) (Figure 2A). To determine the functional importance of the obtained analogous positions, *functional score* distribution was compared against the literature-curated list of driver mutations [from the Cancer Genome Interpreter (CGI)] (50) and benign variants from ClinVar. The overall *functional score* distribution of analogous positions obtained from the analysis showed bimodal distribution with a small proportion of mutations falling within the neutral category (functional score <10). However, the analogous positions showed an enrichment within the non-neutral region (*functional score* >20), comparable to the somatic mutations (statistical hotpots, CGI drivers) and opposed to the distribution scores for benign variants from ClinVar (Figure 2B). Moreover, 48% of the genes with hotspot/resistant positions were within non-CGC genes, in comparison to only 9% analogous positions that were within the CGC genes. Evidently, majority of the positions evaluated by the DOME framework for their involvement in cancer are beyond the known CGC genes. To prioritize the analogous positions, only positions with *functional score* >20 were further considered.

### Catalog of potential driver mutations generated by DOME from all possible missense mutations within the human proteome

**Figure 3.**
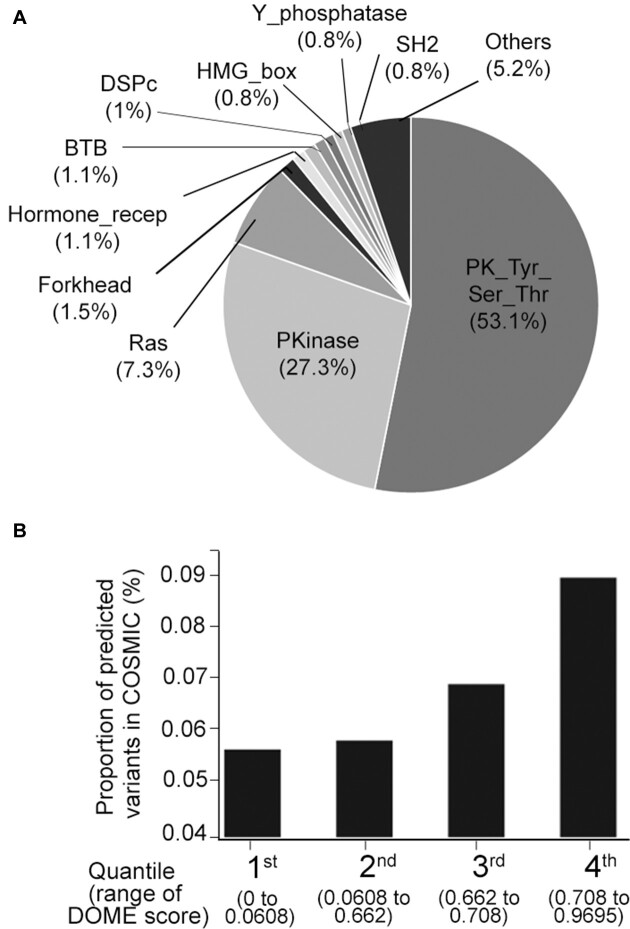
DOME analysis of possible missense mutations within domains across the human proteome. (**A**) Percentage distribution of proportion of DOME predicted drivers falling within the protein domains. (**B**) Overall proportion of mutations designated as somatic in the COSMIC database, across the four quartiles of DOME scores.

To avail the driver mutation position predictions from the complete human proteome as an exploratory resource for easy accessibility, we developed a database with a user interface for querying the analysis results. For this, we first identified all possible analogous positions across the human proteome and prediction scores were generated for all missense variants within the domain. The variants from the CADD database were extracted and prediction scores were generated for 27,904,318 missense variants using the DOME algorithm. The analysis identified 131,671 protein positions that were analogous to statistically significant mutations and have CADD functional score >20 (to filter neutral/population variants). The distribution of the DOME scores for the analogous positions ranged from 0 to 0.969, with the Q3 defined at 0.708, above which the mutations are categorized as high-confidence predicted mutations (Supplementary Figure S2). Additionally, the 32,917 mutations scored in the top quartile range (DOME score 0.70–0.96) by the algorithm were spread across 1331 proteins, 1192 of which were from the non-CGC proteins. The maximum number of predicted low-frequency drivers belonged to the protein tyrosine kinase domain (53%) and protein kinase domain (27%), followed by Ras domain (7%) and others (Figure 3A). The predominance of protein tyrosine kinase and kinase domain protein positions is due to the dominance of hotspot mutation positions within the two domains. Given that the mutations analyzed were mainly theoretical missense variants absent in somatic cancer tissue, we used the presence of these variants in the COSMIC database as a proxy for their potential role or involvement in cancer. The proportion of somatic mutations increased across each quartile of the DOME scores of the predicted driver mutations (Figure 3B). This highlights the DOME scoring framework’s utility in exploring cancer-associated somatic mutations, including novel ones or VUS. The proteome-scale predictions from DOME are made accessible through a GUI for additional exploratory analysis. The GUI enables researchers with no computational know-how to query the protein position of interest (gene search mode) (Supplementary Figure S3A) or run the DOME prediction algorithm on multiple somatic mutations derived from somatic mutation analysis (mutation prediction mode) (Supplementary Figure S3B).

### Benchmarking of the DOME scoring system using *in silico* tools predicting using biochemical information

**Figure 4.**
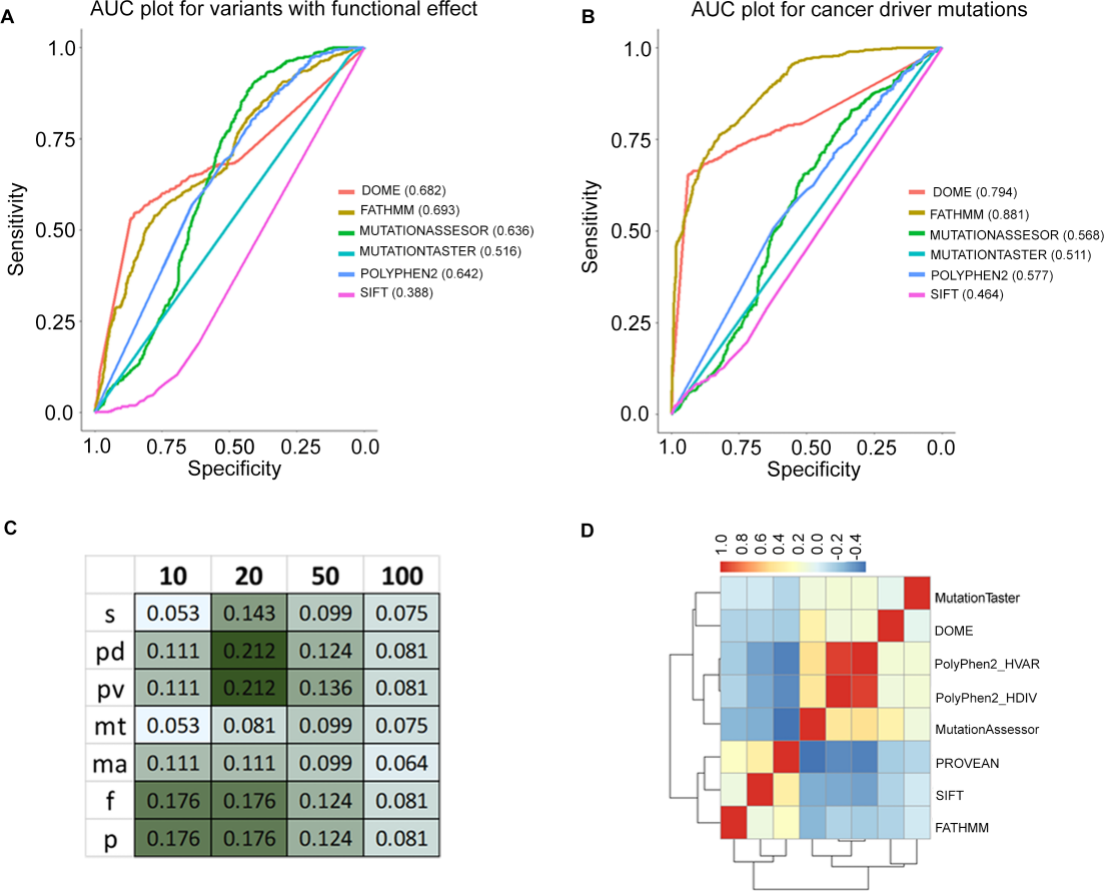
Accuracy evaluation and benchmarking of DOME with the tools in the segment. AUC plot with six different tools for (**A**) functional and (**B**) cancer driver missense mutations reported in the study by Bailey *et al.* (1). The AUCs of the individual methods are mentioned in the legends of the plot next to the tool names. Jaccard coefficient of the TCGA missense mutations (overlapping with the analogous positions), nominated as deleterious by DOME (d), SIFT (s), PolyPhen-2 HDIV (pd), PolyPhen-2 HVAR (pv), MutationTaster (mt), MutationAssessor (ma), FATHMM (f) and PROVEAN (p). Geom-mean indicates geometric mean of the Jaccard coefficient across all the tools. (**C**) Jaccard index between DOME output genes (ranked based on the number of unique TCGA mutations identified as potential drivers); top 10, 20, 50 and 100 gene lists across the tools are compared. (**D**) Pearson’s correlation between the raw prediction scores generated by different tools for individual somatic mutations.

DOME identifies biologically relevant sites with enriched functional scores within protein domains. The framework’s scores were evaluated and benchmarked against seven previously published methods. The seven methods were selected for benchmarking based on similarity of their scoring schemes with DOME, which rely mainly on the biological and biochemical information of the variation, as opposed to recurrence/statistical significance. As a first step, DOME was benchmarked against the missense mutation and its performance to nominate driver variants was evaluated using the reported mutation labels in the study by Bailey *et al.* (1). This study provides two separate sets of annotations, functional and cancer driver annotations. As per the functional mutation annotations, DOME demonstrated an AUC of 0.682, comparable to other tools (Figure 4A). With the cancer driver variants, the AUC of DOME was observed to be 0.794 (Figure 4B), higher than all the other compared tools except FATHMM. To further compare an overlap of driver predictions among seven different tools that use domain/conserved sequence features, we used all the missense variants reported in TCGA (MC3 calls). The comparisons were performed based on the classification of the mutations into ‘deleterious’ and ‘tolerated’ classes by all the tools. Mutations with >0 DOME score were nominated potentially ‘deleterious’; for the rest of tools, the categories were assigned as described in the ‘Materials and methods’ section. DOME was moderately concordant (geometric mean = 0.52) with the deleteriousness classification of the mutations, across the tools. While comparing among the tools, SIFT showed the highest overall concordance (geometric mean = 0.66) in prediction, whereas FATHMM showed the lowest concordance (geometric mean = 0.42) (Supplementary Figure S4). DOME mutation predictions showed highest Jaccard similarity coefficient with MutationTaster, although having altogether different algorithm. DOME scoring tracks purely depend on the biological and biochemical information of the protein position, where MutationTaster assimilates information from biomedical resources and uses naive Bayes classifier to classify mutations (27). Notably, the genes ranked high (based on the number of potential deleterious mutation positions within protein) by DOME substantially differ from those identified by other approaches (Figure 4C). The top-ranking genes identified by DOME (top 10, 20, 50 and 100) have consistently low Jaccard index with sets of genes ranked by alternate methods (Jaccard index <0.25) across all methods. However, relatively higher similarity of DOME is seen with PolyPhen-2 HDIV and HVAR for the top 20 gene list. Overall, low consistency was observed among other tools as well, except for the two versions of PolyPhen-2, which showed highest Jaccard index similarity (data not shown). The same trend is observed in the quantitative correlation analysis of the raw prediction scores among the tools. Overall, DOME demonstrated higher or equivalent accuracy to the existing methods. However, the resulting gene and mutation lists differ significantly from other algorithms, suggesting its complementary role in detecting cancer driver mutations from the cancer genomic dataset (Figure 4D).

### DOME framework identifies potential driver alterations from large-scale somatic mutation datasets

**Figure 5.**
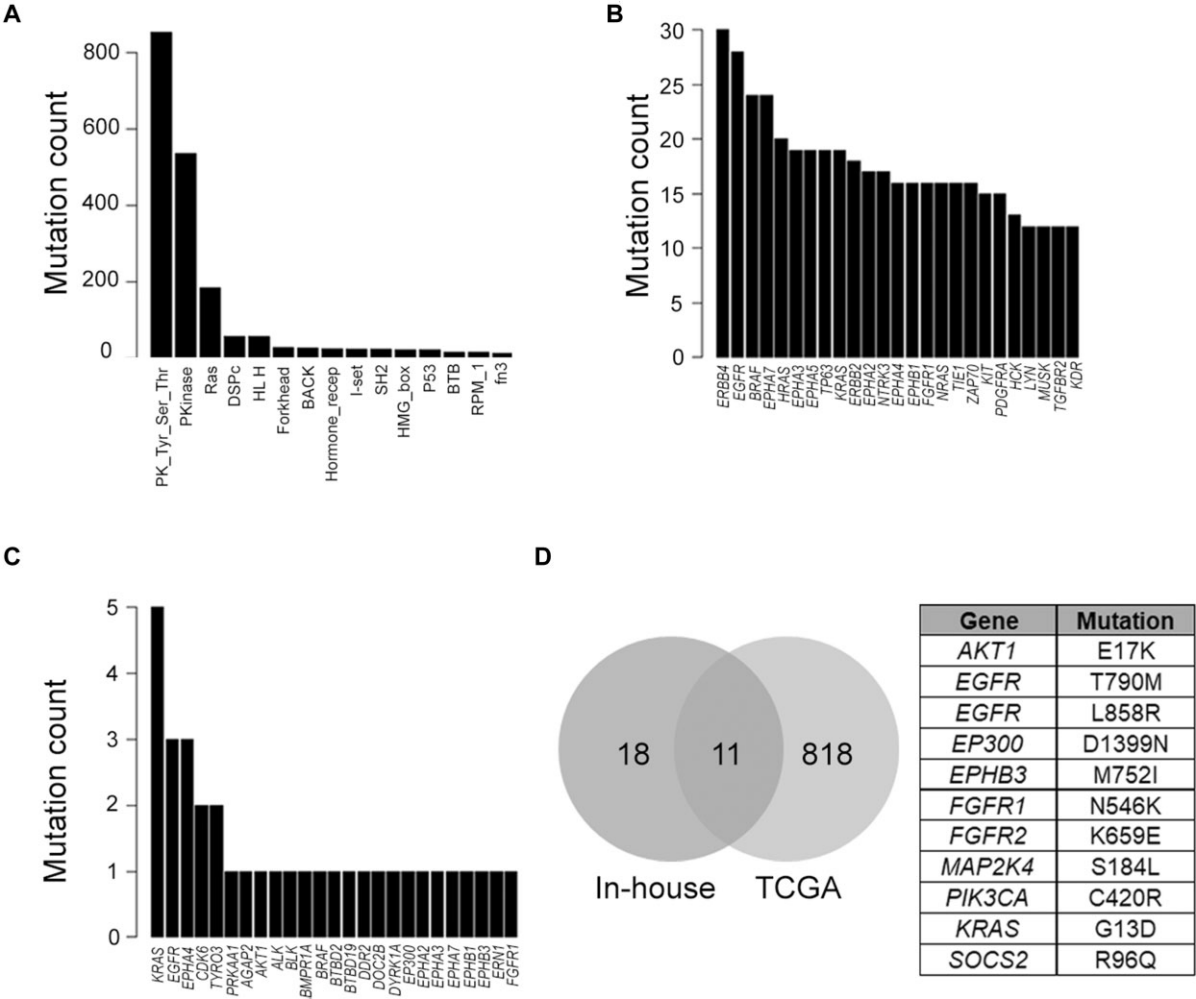
Somatic mutation analysis using the DOME framework for the mutations obtained from large-scale sequencing projects. (**A**) Top 15 domains in which mutations were predicted drivers across TCGA somatic mutations. Top 25 genes with maximum number of analogous positions across TCGA (**B**) and in-house (**C**) somatic mutations predicted to be drivers. (**D**) Overlap between the predicted driver mutations from TCGA and in-house somatic mutations, with the table containing the 11 overlapping mutations.

TCGA somatic mutations downloaded from FireBrowse (firebrowse.org) were reanalyzed using the DOME framework. Of the 827,343 missense mutations from the TCGA pan-cancer dataset, 759,431 (248,314 within the protein domain) were scored by DOME. Among these, 1650 mutations were found in the DOME database showing hotspot/resistant mutation status, representing 901 unique protein positions. Among the mutations within protein domain boundaries, 2364 were found at positions analogous to DOME hotspot/resistant positions, of which 2212 mutations were found to be non-neutral (CADD *functional scores*>20). The non-neutral analogous positions consist of 175 hotspot mutation positions. Among the scored positions, majority of the mutations were found within the protein tyrosine kinase domain (PK_Tyr_Ser-Thr), protein kinase (Pkinase) and Ras domain, followed by others (Figure 5A). All the top 25 genes consisting of the most predicted driver mutations have been assigned the *driver gene* status by the DriverDB3 database and are reported in the literature to be cancer-associated genes. Domains of ERBB4 protein contained the maximum analogous position predicted drivers from the TCGA mutation set, followed by multiple members of the Eph family of receptors, including *EPHA7*, *EPHA3*, *EPHA5*, *EPHA2*, *EPHA4* and *EPHB1* (Figure 5B). As the protein tyrosine kinase domain harbored dominant mutation burden, the top predicted mutations were dominated by their protein members (*n* = 148). A heatmap of prediction scores for the protein tyrosine kinase domain (Supplementary Figure S5) indicates the specific regions in the domain, such as domain region positions 296, 301 and 498. Of these, 296 and 301 constitute structurally critical parts of the protein kinase domain, including HRD region and catalytic region (e.g. corresponding to residues 834 and 841 in EGFR, respectively). Overall, the analysis identified 2035 novel putative driver mutations (1796 listed in the COSMIC database as somatic), of which 762 protein positions were high confidence (score >0.7, median score among all scored mutations by the DOME framework) (Supplementary Table S2).

We further applied DOME for predicting somatic mutations from in-house tumor samples (*n* = 224). To deplete the false-positive germline variants, predominant in Indian population, we filtered the variants reported in TMC-SNPdb 2.0 (42). We identified 29 237 unique somatic missense mutations from the analysis of 224 tumor samples (described in the ‘Materials and methods’ section), of which DOME scores were generated for 26,572 mutations. Concordant to TCGA, 46.7% of in-house somatic mutations mapped within protein domains. DOME analysis flagged 95 mutations as known driver events (as per the driver database) and additionally identified 74 mutation positions as analogous to drivers. Nearly 19% (*n* = 14) of the predicted drivers were found to be known driver mutations flagged in the DOME database. As observed with TCGA analysis, most of the drivers predicted by DOME in the in-house tumors were from the protein tyrosine kinase domain (*n* = 31). The top 25 genes containing maximum number of unique predicted analogous drivers are shown in Figure 5C, wherein different residue variants of 12 and 13 positions of KRAS protein were found to be top of the list. Six members of the Eph protein family members were observed in the top 25 list. Except for EPHA4 (*n* = 3), only singleton events were identified in the other Eph members. A comparison between the high-confidence DOME driver predictions (score >0.7) among the TCGA and in-house tumors resulted in 11 overlapping mutations (Figure 5D), 10 of which have already been characterized as driver mutations across different cancer types, and additionally predicts mutation in protein EPHB3 (752, M > I) as a potential driver mutation. The mutations predicted to be potential drivers, along with the scores, are provided in Supplementary Table S3.

## Discussion

Several analytical methods contribute to the identification of driver genes involved in tumorigenesis, which highlight the enrichment of somatic mutations at specific genomic loci (51,52). While some rely on the statistical enrichment and clustering of mutations within regions (53), others evaluate the functional impact of mutations (54). These methods are built upon diverse layers of biological information, including enrichment in post-translational modifications (55,56), 3D geometric structures (57–59), cell signaling pathways (56,60–62), interaction interfaces (58,63) and protein networks (60,61,64).

Current methods for analyzing large-scale genomic datasets, such as TCGA and the International Cancer Genome Consortium, have underlined a critical need for methods to detect low-frequency mutation events and their mechanistic impacts (65). Driver mutation discovery is complicated by biological context (disease stage, microenvironment, immune status), secondary clonal expansion during therapy response and ethnic variations. Any typical somatic mutation analysis generates a tail of several nonrecurrent mutations that are classified as VUS, but potentially may contribute to disease progression in specific context.

In this work, we introduce an analytical framework, DOME, to predict potential driver mutations from tumor-specific variation datasets. This framework is designed to complement the current recurrence-based statistical approaches and aimed to identify potential driver mutations from the mutations that recur poorly. A combined catalog of 1527 known (literature/database-derived) driver/therapy-resistant mutations, along with statistical assessment of recurrent somatic mutations from 7541 patient samples representing 33 tissues in TCGA, was performed for individual tumor types. Mapping the 1527 mutations on proteins with multiply aligned domains, 32,099 positions were derived based on residue conservation spanning 83 unique protein domains. Since we hypothesize that the conserved residue positions (analogous) across proteins within the domain that are aligned to the statistically significant mutation positions may have similar functional consequence and may play a role in cancer, we performed *in silico* assessment by comparing their functional (CADD) score distribution to literature-curated drivers, and ClinVar-derived benign variants as control. The functional score distribution of analogous residue protein positions was found in agreement with driver mutation residue scores (Figure 2B). We developed a scoring scheme to rank the mutations analyzed. The scoring algorithm integrates the domain conservation, biochemical/functional site context, somatic residue-change propensities and distribution of mutations across proteins having same domain (entropy), further using population neutrality as a filter to reduce the false-positive driver predictions. The DOME algorithm was applied to analyze somatic mutation data from TCGA and 224 in-house Indian patient samples representing seven cancer types. The tyrosine kinase domain, consisting of >100 protein members, harbored majority of statistically significant mutations (Figure 5A). Analysis highlighted known high-frequency mutations in *EGFR*, *BRAF*, *ERBB4* and *HRAS*, as well as multiple low-frequency variants in the Eph family of proteins (*EPHA7*, *EPHA3*, *EPHA5*, *EPHA2*, *EPHA4* and *EPHB1*). Eph family members contribute to developmental pathways, including segmentation, migration, angiogenesis and axon guidance (66). In cancer, Eph family members show a context-specific role as tumor promoters or suppressors and are associated with specific cancer hallmarks (67–69). Mutations in Eph family of proteins have been observed in the metastatic colorectal tumors, which are predicted to be loss-of-function events for some of these tumor suppressor proteins (70,71). Like the TCGA mutations, multiple Eph protein family members were found to contain predicted driver mutations (Figure 5B). An earlier unreported missense mutation in *EPBH3* protein at 752 sequence position (AA change M→I) was identified in the high-confidence list of predicted drivers from both in-house sample and TCGA dataset. *EPHB3* has been reported to have a tissue-specific role, acting as a tumor suppressor in colorectal cancer (72,73), head and neck squamous cell carcinoma (HNSC) (74) and metastases, or proliferation promoting factor in non-small-cell lung and papillary thyroid cancer in a kinase activity-dependent manner (75,76). *EPHB3* co-amplification along with *PIK3CA* is reported in HNSC samples (74). However, frequent mutations are not reported in any cancer type. Identification of functional relevance of Eph family mutations warrants further functional exploration, as members of this family have been implicated as targets in cancer therapy (77).

The benchmarking results highlight variations in prediction outcomes between the DOME framework and seven different tools, including widely adopted methods such as SIFT. A prediction accuracy evaluation of DOME for variants in the study by Bailey *et al.* (1) demonstrates that our approach reaches a prediction accuracy (AUC) of 0.68 for the functional variants (comparable to most other tools) and 0.79 for cancer drivers, respectively. Notably, the FATHMM algorithm performs best followed by DOME, against the annotated cancer drivers among the compared methods. A commonality between DOME and FATHMM is that both make inferences about driver nature of mutation directly from protein domain information, once again highlighting specific protein domains in cancer. Although DOME exhibits moderate concordance in predicting driver genes, particularly with similarities to MutationTaster, it distinguishes itself by consistently identifying well-established hotspots and demonstrating a unique capacity for predicting nonrecurrent mutations. This distinctive capability underscores the complementarity of the DOME framework within the broader landscape of driver assessment methods. Our benchmarking underscores the diversity of approaches within the field of cancer mutation analysis. It is important to note that although DOME and FATHMM outperform other methods in predicting specifically cancer drivers, overlap between the driver genes predicted among these is low (Figure 4C), suggesting that both the methods highlight distinct mutation set as per their respective metrics. It is important to acknowledge that DOME exhibits lower concordance with other tools. However, it is evident that analogous mutation positions identified by DOME show similar functional score distribution to hotspot (known) drivers (Figure 2B) and DOME scores linearly show enrichment of cancer-associated mutations (Figure 3B), suggesting its ability to identify novel driver mutations. This also underscores the utility of DOME when used in addition to the common cancer driver prediction methods. We also conducted proteome-wide mutation predictions for all possible missense mutations derived from the CADD database. Of all the mutations analyzed, scores were provided for 131,671 missense mutations by DOME. As this theoretical whole proteome missense variant dataset is agnostic of any association with cancer, we assessed the enrichment of somatic mutations for the different quartiles of DOME scores. There was an incremental increase in the proportion of COSMIC reported mutations with increase in the DOME scores (Figure 3B). Hence, concordance of DOME score was observed with the functional residue scores, as well as with reported cancer-associated mutations. The algorithm and the database are made available as a part of a GUI toolkit. The toolkit is designed for easy integration of DOME into the existing somatic analysis pipelines and allows accessing specific mutations in the proteins (gene position search mode) and performing predictions on the user-defined somatic mutation dataset (somatic mutation search mode), arising from a tumor-specific analysis (Supplementary Figure S3). The downloadable Python package of DOME also allows researchers to update the background statistical significant mutation and computes analogous mutations that allow extensions of the in-built database. The main limitation of this approach is that DOME predictions are limited to missense mutations within protein domains, excluding evaluation of other mutations outside the domain boundaries. Also, the domain boundaries considered in the study are based on the PFAM database that may have resulted in rare exclusion of the mutations falling within UniProt database domain boundaries (based on the Prosite rule), such as *PIK3CA* (1047, H→R), a known therapeutically relevant mutation (78). We also note that DOME performs scoring referring to the canonical transcripts and oblivious to the shifting domain positions in the alternate isoforms. Lastly, it is important to recognize that the field of low-frequency driver mutation identification methods, including DOME, faces a fundamental challenge in the absence of a gold standard mutation dataset for validation. In the absence of an extensively curated rare (nonrecurrent) somatic mutation dataset, DOME predictions remain a valuable initial step in highlighting potential candidates, but their true significance awaits validation through functional genomic studies, to assess their role in cancer progression and therapeutic implications. Although we believe that the analytical framework presented in this study is dynamic and with inclusion of wider reference hotspot or founder datasets, it can be applied to broader analytical aspects of cancer, such as synthetic lethality, clonal mutagenesis and prediction of targeted therapy resistance, among others. DOME is also likely to be a promising approach in addressing VUS in personalized cancer diagnostics.

In conclusion, explosion of the large-scale tumor sequencing project has created a need of novel methods to interpret and annotate the long tail of low-frequency somatic mutations, which are ignored in the frequentist methods for driver discovery. Here, we present a computational approach to identify putative driver positions of conserved residues within protein domains. This approach has the potential to identify mutations that are not detected by other methods, as well as to provide insights into their functional and biochemical consequences. Thus, DOME complements and extends the current approaches to aid in discovery of novel, low-frequency, therapeutically actionable mutations to advance our understanding of the genetic basis of cancer, and ultimately to develop new strategies for diagnosis and treatment. By leveraging the power of genomic sequencing and computational tools like DOME, clinicians may also be able to identify and interpret VUS, which poses a significant challenge to personalized cancer diagnostics more effectively, leading to more personalized and effective cancer treatments for patients.

## Supplementary Material

zcae010_Supplemental_Files

## Data Availability

The DOME algorithm and GUI-enabled desktop application are freely available for download for the academic users at https://www.actrec.gov.in/piwebpages/AmitDutt/DOME/DOME.html. The code is available at https://doi.org/10.5281/zenodo.10672766.
