# Supplementary material for: Singleton mutations in large-scale cancer genome studies: uncovering the tail of cancer genome
Source: NAR Cancer. 2024 Mar 12;6(1):zcae010. doi: 10.1093/narcan/zcae010 (PMC10939354; doi:10.1093/narcan/zcae010)
Supplement: zcae010_Supplemental_Files [file zcae010_supplemental_files.zip › Supplementary-figures.pdf]

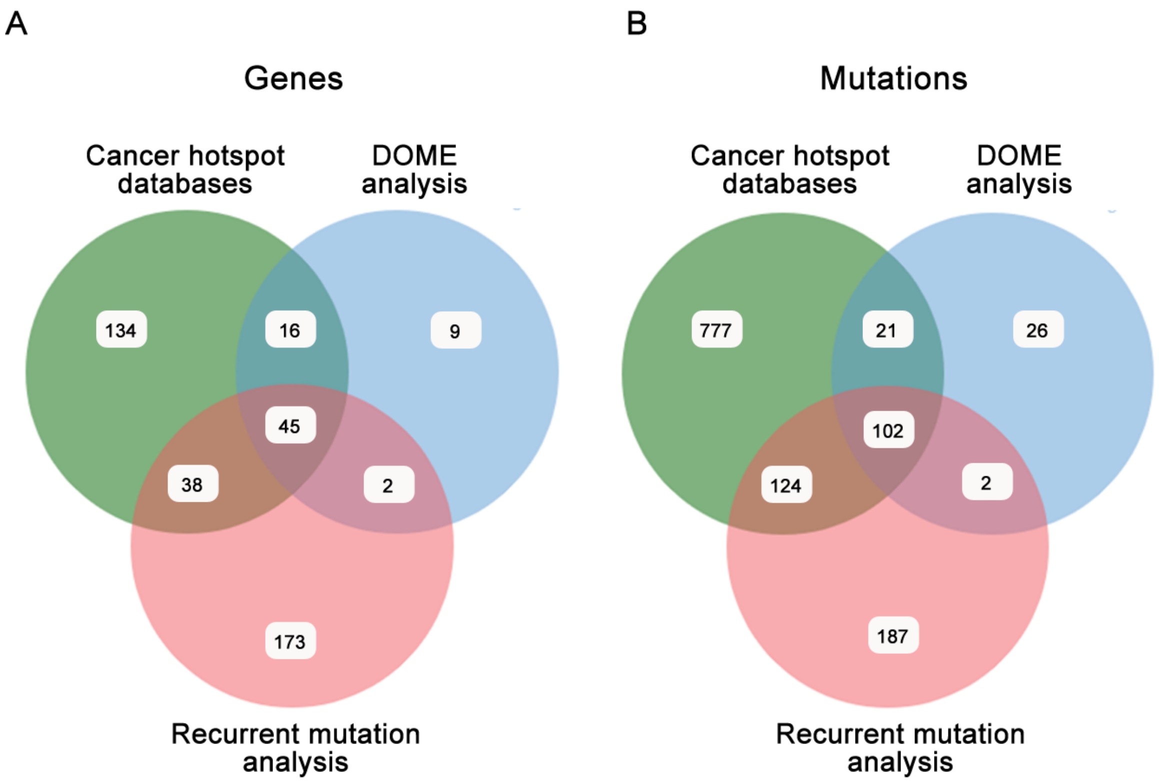

Figure S1: Gene (A) and mutation (B) -wise overlap between the cancer hotspot database (cancerhotspots.org), the binomial based hotspot identification performed in the current study and literature reported recurrent drivers

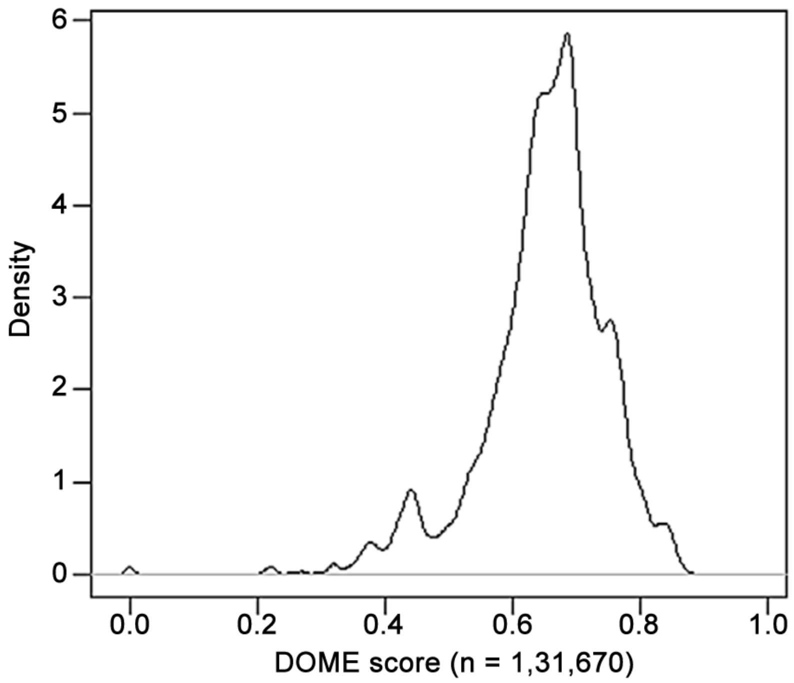

Figure S2: Distribution of scores for analogous positions scored by DOME, for all possible missense mutations across human proteome within domains

A

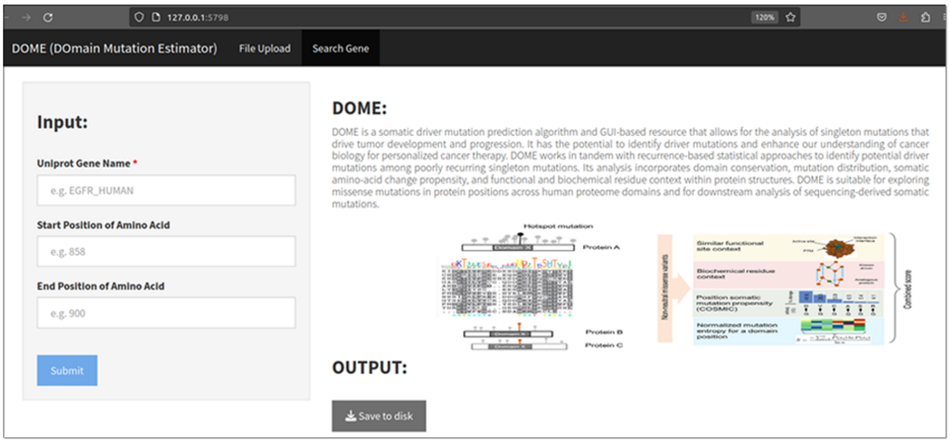

B

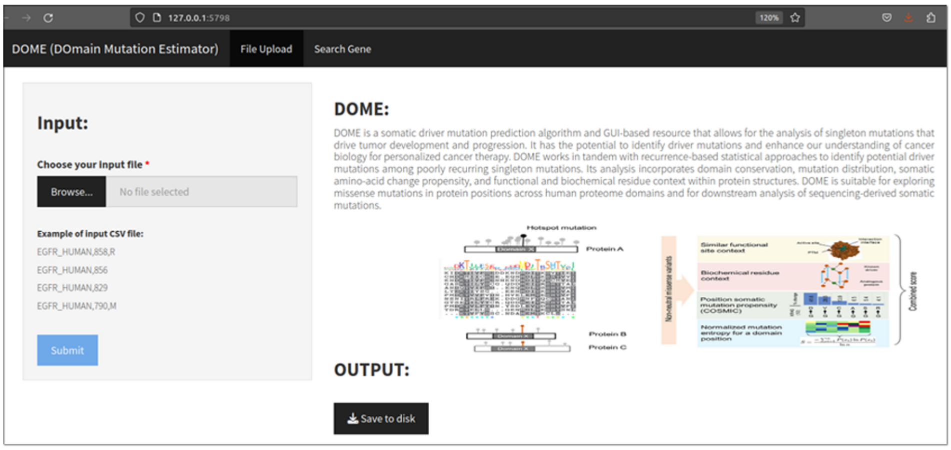

Figure S3: Graphical User Interface to individually access, query and execute DOME on the somatic mutation datasets in two modes; (a) gene search mode – allowing a gene wide position search, (b) mutation score prediction mode – allows assignment of DOME score to somatic mutations in the VCF query file.

	d	s	pd	pv	mt	ma	f	Geom mean
**DOME**								0.525
**SIFT**	0.556							0.666
**Polyphen2_HDIV**	0.549	0.746						0.655
**Polyphen2_HVAR**	0.540	0.740	0.938					0.649
**MutationTaster**	0.610	0.732	0.786	0.749				0.635
**MutationAssessor**	0.518	0.559	0.540	0.555	0.494			0.500
**FATHMM**	0.383	0.458	0.437	0.433	0.447	0.339		0.427
**PROVEAN**	0.549	0.847	0.726	0.715	0.717	0.560	0.458	0.658

Figure S4: Benchmarking using Jaccard coefficient (JC) of the TCGA missense mutations (overlapping with the analogous positions), nominated as deleterious by DOME (d), SIFT (s), Polyphen-2 HDIV (pd), Polyphen-2 HVAR (pv), MutationTester (mt), MutationAssessor (ma), FATHMM (f) and PROVEAN (p). Geom-mean indicates geometric mean of the JC's across all the tools.

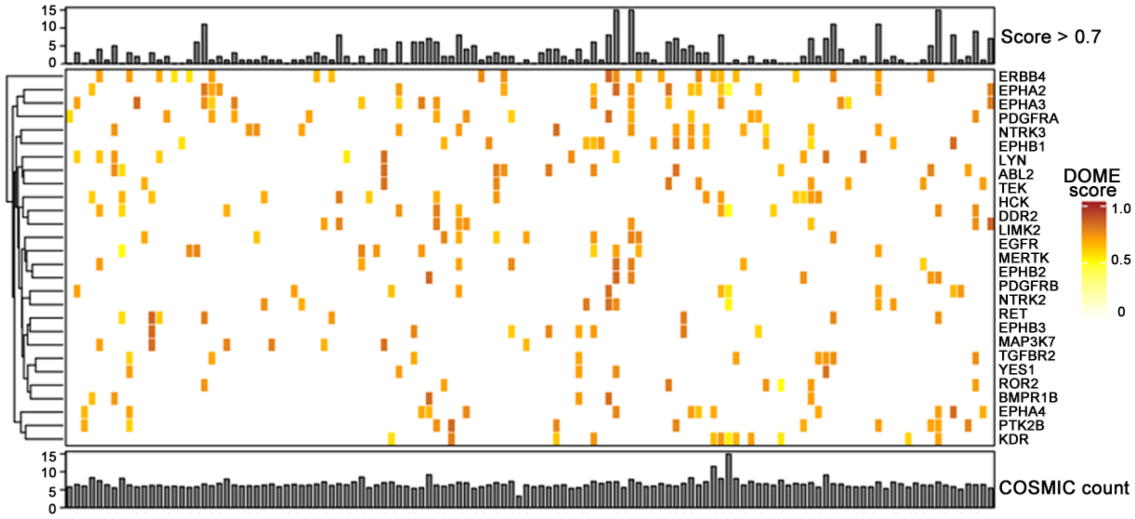

Figure S5: Heatmap representation of the scores for the mutated residues within the tyrosine kinase protein domain in TCGA
